# Impact of Restriction-Resumption Protocols on Mood and Anxiety in Healthy Adults: Randomized Controlled Trial

**DOI:** 10.2196/90532

**Published:** 2026-05-20

**Authors:** Nickolai Titov, Alison Dagnall, Alana Fisher, Olav Nielssen, Rony Kayrouz, Michael Jones, Heather Hadjistavropoulos, Daniel Rock, Lauren Staples, Blake Dear

**Affiliations:** 1 Macquarie University Sydney, New South Wales Australia; 2 University of Regina Regina, SK Canada; 3 Psychiatry, Medical School The University of Western Australia Perth, Western Australia Australia; 4 WA Primary Health Alliance Perth, Western Australia Australia; 5 Health Research Institute Faculty of Health University of Canberra Canberra, ACT Australia

**Keywords:** depression, anxiety, deterioration, restriction, resumption, prevention, mechanisms, behavior, cognitions, habits, etiology, psychological model, disability, methodology

## Abstract

**Background:**

Simple behavioral and cognitive actions can reduce symptoms of anxiety and depression. However, there is limited research investigating whether restricting those same actions increases symptoms and whether resuming those actions reduces symptoms.

**Objective:**

The primary aim was to examine the impact of restricting, then resuming (the “restriction-resumption protocol”), 5 groups of actions called the Things You Do (TYD), on symptoms of depression and anxiety. Participant satisfaction with life and perceptions of changes in behavior and mental health were also evaluated.

**Methods:**

In total, 70 adults were randomly allocated to an intervention group (IG) or a control group (CG). IG participants completed a 3-phase protocol over 8 weeks: a 2-week baseline period (phase A), 2 weeks of restricted TYD actions (phase B), and a 4-week resumption phase (phase C). CG participants were instructed to maintain their usual activities and routines. Primary outcomes included symptoms of depression (Patient Health Questionnaire–9), anxiety (Generalized Anxiety Disorder–7), and frequency of TYD actions. Secondary outcomes included satisfaction with life and perceived changes in mental well-being. Outcomes were measured weekly throughout the 8-week trial and again after the trial at week 9.

**Results:**

Scores on outcome measures did not differ between groups during phase A and after the trial. However, by the end of phase B, IG participants showed significantly increased symptoms of depression and anxiety compared to CG participants (*P*s<.001). Large within-group effect sizes were observed on the Patient Health Questionnaire–9 and Generalized Anxiety Disorder–7 from baseline to the end of phase B (Cohen *d*>1.8) and from phase B to after the trial (Cohen *d*>1.9). Changes in satisfaction with life and evaluations of mental health corresponded with symptom changes.

**Conclusions:**

These findings demonstrate a relationship between the frequency of performing specific actions and symptoms of depression and anxiety. The restriction-resumption protocol has the potential to increase our understanding of causal mechanisms underlying common mental disorders and may lead to the development of new models of prevention and treatment.

**Trial Registration:**

Australian and New Zealand Clinical Trials Registry ACTRN12624001491550; https://www.anzctr.org.au/Trial/Registration/TrialReview.aspx?id=388828&isReview=true

## Introduction

Depressive and anxiety disorders are common and disabling, but many people choose not to access treatment [1], and not all individuals who receive treatment experience improvement [2]. Studies have demonstrated that increasing the frequency of simple behavioral and cognitive actions, such as performing meaningful activities [3], regular exercise [4], and practicing kindness [5], can reduce symptoms [6,7]. However, it is not known whether some actions confer greater benefits than others, or how frequently they need to be performed to produce a meaningful effect. In a series of studies, we sought to answer these questions. First, using a cross-sectional survey design, we found that 5 out of 16 groups of actions had the strongest relationship with symptoms of depression and anxiety in a large sample of adults [8]. The “Things You Do” (TYD) are (1) realistic thinking, (2) meaningful activities, (3) having goals and plans, (4) healthy habits and routines, and (5) regular social contact. Building on previous research [9-11], we demonstrated that performing the actions at least half the days of each week was a threshold for measurable benefit [8,12]. Subsequent studies using longitudinal designs found a linear relationship between the frequency of performing the TYD actions and changes in symptoms of depression and anxiety in large samples of Australian [13] and Canadian [14] adults seeking psychological treatment. Those studies demonstrated that, as treatment progressed, the frequency of performing the TYD actions increased as symptoms reduced. Recently, a self-guided intervention comprising an education module about the TYD actions and daily email prompts for 4 weeks encouraging people to perform the actions resulted in moderate to large reductions in symptoms of depression and anxiety [15]. In a randomized controlled trial (RCT) consisting of a similar brief TYD intervention with SMS text messaging prompts, symptoms of depression and anxiety significantly reduced in the intervention group (IG) compared to a waitlist control group (CG) [16].

A small pilot trial (n=12) designed to further test the relationship between TYD actions and mental well-being reported that restricting the frequency of TYD actions led to a significant increase in symptoms of depression and anxiety in asymptomatic volunteers, which then resolved when they resumed those actions [17]. The primary aim of this study was to confirm these results using an RCT design; that is, whether systematically restricting and then resuming the TYD actions in asymptomatic volunteers led to an increase and then a decrease in symptoms of depression and anxiety in healthy adults. The secondary aim was to explore participants’ awareness (or perceptions) of changes in their behavior and mental health. We hypothesized that, relative to a CG, participants who restricted the TYD actions would experience mild to moderate increases in symptoms of depression and anxiety, which would return to baseline levels after they resumed those actions.

## Methods

### Study Design

A 2-arm parallel-group RCT design was used, with participants randomly stratified by gender and age and allocated to an IG or CG by an investigator (BD) who was not involved in recruitment. Allocations were concealed until applicants were deemed eligible during a telephone interview.

On the basis of our previous methodology [17], the restriction-resumption protocol followed by the IG involved 3 phases over 8 weeks: phase A (2-week baseline), phase B (2-week restriction), and phase C (4-week resumption). The CG completed questionnaires at the same time as the IG, but participants were instructed to maintain their usual routines and actions. We estimated that detecting a reliable between-group effect size of d ≥0.80 at the end of phase B (*P*<.05; power=0.80; 1-tailed) would require a minimum sample size of 21 participants per group [17].

### Ethical Considerations

The study was approved by the Macquarie University Human Research Ethics Committee (520241769358699) and prospectively registered on the Australian and New Zealand Clinical Trials Registry (ACTRN12624001491550). Applicants were informed that one study condition required participants to restrict actions that could lead to increased symptoms of depression and anxiety. Consent was obtained and recorded digitally (online) at baseline from all participants. Given the nature of the IG condition, consent was also reaffirmed verbally each week phases B and C of the trial. The study website and the Participant Information and Consent Form indicated that participants would receive partial compensation for their time in the form of gift vouchers, with a maximum total value of Aus $250 (US $167.54). The privacy and confidentiality of participants were maintained throughout the study.

### Participants

The study was conducted via an online university clinical trials unit and promoted via the clinic’s social media and website, as well as through a participant recruitment company. People wishing to apply completed an online screening assessment comprising questionnaires assessing their symptoms and other information.

The inclusion criteria were (1) age ≥18 years, (2) a score of <10 on the Patient Health Questionnaire–9 (PHQ-9) [18] and a score of <8 on the Generalized Anxiety Disorder–7 (GAD-7) [19], and (3) Australian residency. Exclusion criteria were (1) inability to read and understand English and (2) current receipt of psychological treatment.

Applicants participated in a structured telephone interview with a registered mental health professional (NT or AD), during which eligibility was confirmed. Applicants were enrolled after they had confirmed that they would follow the restriction instructions should they be allocated to the IG.

### Primary Measures and Time Points

The 3 primary measures were administered weekly. Outcomes were compared at 3 primary time points: week 1 (baseline), week 5 (end of the restriction phase), and week 9 (posttrial phase).

The PHQ-9 was used to measure depressive symptoms congruent with the Diagnostic and Statistical Manual of Mental Disorders, fourth edition (DSM-IV) over the previous 2 weeks. Total scores range from 0 to 27, with scores ≥10 indicating a likely clinical diagnosis of depression. The PHQ-9 has good internal consistency and is sensitive to change, and scores can be categorized as healthy (0-4), mild (5-9), moderate (10-14), moderately severe (15-19), and severe (20-27) [18]. Cronbach α in this trial was 0.72 at week 1.

The GAD-7 was used to measure general anxiety symptoms over the previous 2 weeks. Total scores range from 0 to 21, with scores ≥8 indicating DSM-IV–congruent generalized anxiety disorder. The GAD-7 is also sensitive to social phobia and panic disorder. Scores are categorized as healthy (0-4), mild (5-7), moderate (8-14), and severe (15-21) [20,21]. Cronbach α at week 1 was 0.72.

The TYD Questionnaire–15 (TYDQ-15) [13] measures actions associated with good psychological health and comprises 3 actions from each of the 5 subscales of the TYD [8]. The 5 subscales include healthy thinking, meaningful activities, goals and plans, healthy habits, and social connections (Multimedia Appendix 1). Participants used a 5-point Likert rating scale to indicate how often they performed each action over the previous week: 0=“Not at all,” 1=“One or two days,” 2=“Half the days,” 3=“Almost every day,” and 4=“Every day.” Scores on the TYDQ-15 range from 0 to 60. Cronbach α at week 1 was 0.91.

### Secondary Measures

Two secondary measures were administered: the Satisfaction With Life Scale (SWLS) [22] and a bespoke questionnaire, the Reflections Questionnaire (RQ). The SWLS consists of 5 items using a 7-point Likert scale ranging from 1 (“Strongly disagree”) to 7 (“Strongly agree”). Total scores range from 5 to 35, with higher scores indicating greater life satisfaction. The SWLS was administered at assessment; at weeks 1, 3, and 5; and after the trial. Cronbach α at week 1 was 0.88.

The RQ comprised 3 questions. The first question asked participants whether they perceived changes in their mental health in the previous week, with 7 categorical response options ranging from “greatly improved” to “greatly deteriorated.” The second and third questions asked participants to describe reasons for any observed mental health changes and to describe anything they had learned about their mental health in the previous week. A thematic analysis of the qualitative responses to questions 2 and 3 of the RQ has been reported in a separate study [23].

### Procedure

IG and CG participants began the trial at the same time. During phase A of the restriction-resumption protocol, the IG participants were instructed to maintain their usual activities. During phase B, they were instructed to restrict the frequency of performing the actions listed in the TYDQ-15 to ≤2 times per week, a threshold previously identified as associated with poor mental health [8]. These directions were provided via online materials, which included a checklist (Multimedia Appendix 2), and a telephone call by a study clinician (NT or AD) either immediately before the end of phase A or at the start of phase B. Participants also received a weekly email reminding them of the relevant phase. During phase C, IG participants received online materials instructing them to increase the frequency of performing the actions listed in the TYDQ-15 to ≥5 times each week or back to their usual levels (Multimedia Appendix 3). Participants also received an online information module describing the importance of the TYD actions, along with 1 SMS text message each weekday at 10 AM Australian EST encouraging them to perform the target actions. Participants received 1 SMS text message from each of the 5 categories of actions each week (Multimedia Appendix 4). IG participants were telephoned weekly to monitor well-being and address any questions.

### Statistical Analysis

Data were analyzed using SPSS (version 29; IBM Corp). A significance level of .05 was used for all tests. Group differences at assessment were examined using chi-square analysis for categorical variables and ANOVA for continuous variables. Changes over time in the continuous variables (TYDQ-15, PHQ-9, GAD-7, and SWLS) were analyzed for participants who started the trial. To evaluate the statistical significance of group-by-time effects, generalized estimating equations using a gamma log-link function and an unstructured working correlation matrix were used. Pairwise comparisons identified significant within-group changes across phases. Between-group differences in TYDQ-15 scores at each time point were examined to demonstrate adherence to the protocol. Cohen *d* effect sizes were used to evaluate the clinical significance of symptom changes, and reliable changes on the primary measures were also calculated using the reliable change index [24]. In this study, the reliable change index was ≥9 for the TYDQ-15, ≥4 for the PHQ-9 and GAD-7, and ≥5 for the SWLS. Reliable change in response to restriction was calculated from baseline (week 1) to the end of restriction (week 5). Reliable change in response to resumption of activities was calculated from week 5 to week 9. Reliable change from week 1 to week 9 was calculated to measure overall change and to confirm return to baseline. Chi-square analysis was used to examine categorical differences on question 1 of the RQ.

## Results

### Participant Characteristics

**Figure 1:**
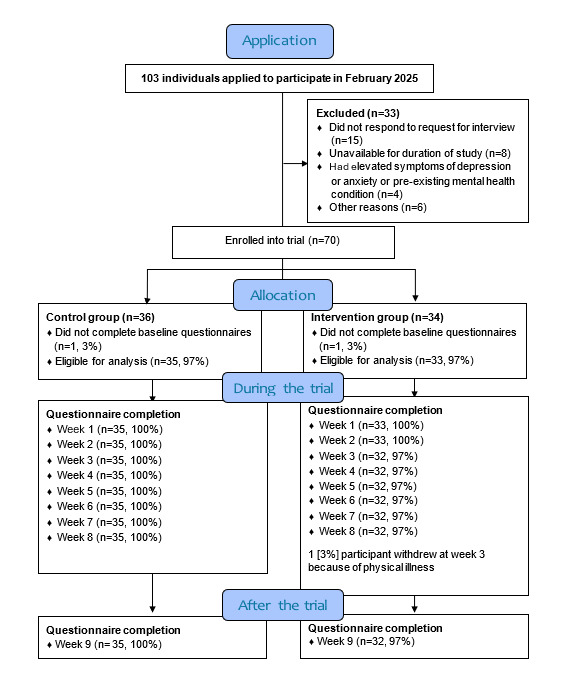
CONSORT (Consolidated Standards of Reporting Trials) 2010 flow diagram.

Between February 3, 2025, and February 21, 2025, a total of 103 individuals consented and applied. Of these, 33 (32%) applicants were ineligible, and 70 (67.9%) applicants met all inclusion criteria and were enrolled and randomized (Figure 1).

In total, 36 participants were allocated to the CG, and 34 participants were allocated to the IG. One person in each group did not start and was not eligible for analysis. One IG participant withdrew in week 2 due to the onset of a physical illness unrelated to the trial. Missing values for this participant were not replaced. With this exception, questionnaire completion rates were 100%.

Demographics are shown in Multimedia Appendix 5. Randomization was mostly successful, except that IG participants had higher baseline levels of education (*χ*²_2_=11.2; *P*=.004). A minority of the overall sample (14/68, 20.6%) reported “very mild” or “mild” difficulties with anxiety; all participants reported no current difficulties with depression and no use of medication for mental health reasons. A minority of the overall sample (9/68, 13.2%) reported previous consultations regarding their mental health, and all confirmed that they were not currently receiving psychological therapy. Mean total scores at enrolment on the PHQ-9 were 1.0 (1.6) and on the GAD-7 were 0.6 (1.2), which were both in the healthy range (<5) (Table S1 of Multimedia Appendix 5).

### Adherence to the Restriction-Resumption Protocol

Mean scores at each week are shown in Table 1. IG participants’ scores on outcome measures and responses to question 2 of the RQ showed no evidence of deviation from or breach of the protocol for most IG participants (30/33, 90.9%). In total, 3 (9.1%) of the 33 participants had an increase in PHQ-9 and GAD-7 scores and a decrease in TYDQ-15 scores in the week before phase B began. When asked about this, 2 (6.1%) reported that they began restricting activities a week earlier than instructed, for reasons that were unclear. The other 1 participants reported experiencing a physical illness, which they said was responsible for the observed changes.

**Table 1:** Means, SDs, and effect sizes by group and week.

Measure			Means (SDs)																			Measure and group, Cohen *d* (95% CIs)				
			Week 1		Week 2		Week 3		Week 4		Week 5		Week 6		Week 7		Week 8		Week 9		Week 1 to week 5			Week 5 to week 9		Week 1 to week 9
**Things You Do Questionnaire–15**																										
	CG^a^	44.5 (9.7)		45.4 (10.2)		47.0 (10.2)		47.4 (9.8)		47.4 (9.2)		45.4 (8.2)		48.6 (9)		48.8 (9.6)		49.3 (9.4)		0.31 (−0.17 to 0.77)			0.20 (−0.27 to 0.67)		0.50 (0.02 to 0.97)	
	IG^b^	45.8 (8.5)		46.6 (8.5)		43.5 (13.7)		18.4 (9.6)		18.2 (8.6)		41.3 (8.3)		46.4 (8.7)		49.2 (8)		49.4 (9)		−3.23 (−3.92 to −2.46)			3.54 (2.72 to 4.28)		0.41 (−0.08 to 0.90)	
**Patient Health Questionnaire–9**																										
	CG	2.3 (2.8)		2.1 (2.9)		1.7 (2.3)		1.7 (2.6)		1.4 (1.9)		1.3 (1.9)		0.9 (1.4)		0.9 (1.7)		1.0 (1.4)		−0.38 (−0.84 to 0.10)			−0.24 (−0.71 to 0.23)		−0.59 (−1.06 to −0.10)	
	IG	1.5 (1.4)		1.5 (1.6)		1.8 (2.9)		7.0 (4.6)		6.9 (3.4)		2.4 (2.1)		1.2 (1.5)		1.2 (1.7)		0.9 (1.3)		2.09 (1.46 to 2.67)			−2.33 (−2.93 to −1.67)		−0.44 (−0.93 to 0.05)	
**Generalized Anxiety Disorder–7**																										
	CG	1.4 (2.1)		1.2 (1.6)		1.2 (2.3)		1.1 (1.7)		0.9 (1.5)		1.0 (1.7)		0.9 (1.4)		0.9 (1.7)		0.7 (1.3)		−0.27 (−0.74 to 0.20)			−0.14 (−0.61 to 0.33)		−0.40 (−0.87 to 0.08)	
	IG	1.2 (1.5)		1.1 (1.6)		1.5 (2.7)		5.9 (4.3)		5.6 (3)		2.2 (2.2)		1.2 (1.9)		0.9 (1.6)		0.8 (1.7)		1.86 (1.26 to 2.42)			−1.97 (−2.54 to −1.35)		−0.25 (−0.73 to 0.24)	
**Satisfaction With Life Scale^c^**																										
	CG	26.5 (5.1)		—^d^		27.1 (4.7)		—		27.2 (4.6)		—		—		—		27.4 (4.5)		0.14 (−0.33 to 0.61)			0.04 (−0.43 to 0.61)		0.19 (−0.28 to 0.65)	
	IG	27.0 (4.8)		—		26.5 (5.6)		—		22.4 (6.3)		—		—		—		28.2 (4.3)		−0.82 (−1.32 to −0.31)			1.08 (0.54 to 1.59)		0.26 (−0.23 to 0.75)	

^a^CG: control group.

^b^IG: intervention group.

^c^The SWLS was not administered at week 2, 4, or 6-8.

^d^Not applicable.

There were no between-group differences in TYDQ-15 scores at baseline (week 1: *F*_1,66_=0.364; *P*=.55) or after the trial (week 9: *F*_1,66_=0.002; *P*=.97). IG participants had significantly lower TYDQ-15 scores than CG participants at the end of restriction (*P*<.001), with TYDQ-15 scores approximately 41% of the baseline scores. These results indicate overall IG compliance with the protocol.

### Changes Over Time on the Primary Measures

**Figure 2:**
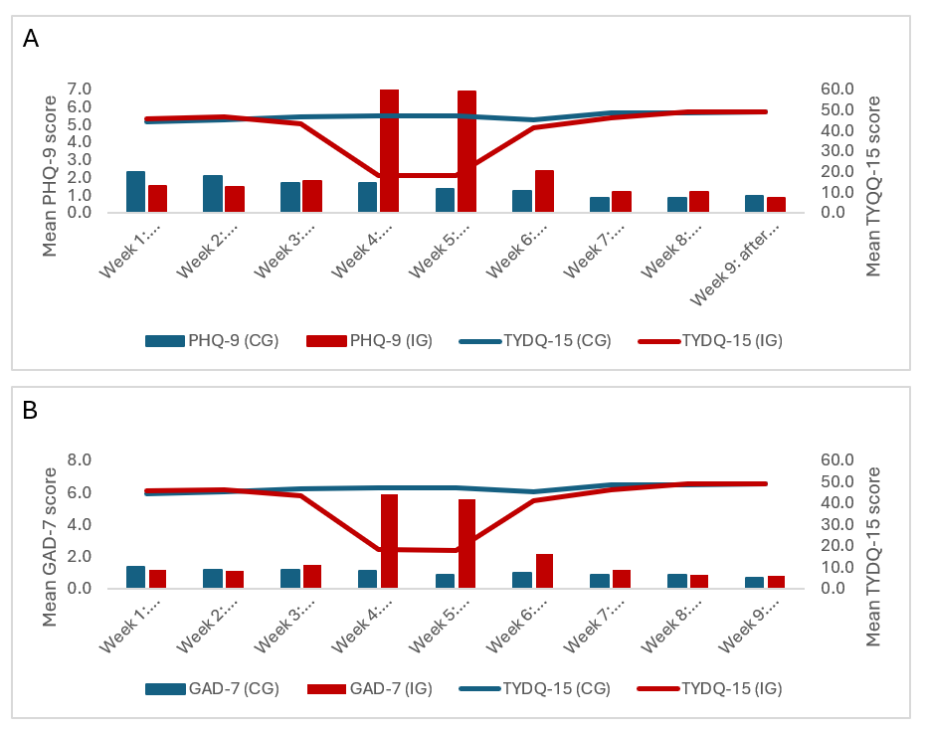
Changes over time on the Things You Do Questionnaire–15 (TYDQ-15) compared with (A) the Patient Health Questionnaire–9 (PHQ-9) and (B) the Generalized Anxiety Disorder–7 (GAD-7). CG: control group; IG: intervention group.

Means and effect sizes are shown in Table 1. For the IG, large within-group effect sizes were observed on the primary measures from baseline to the end of restriction (Cohen *d*>1.8) and from restriction to after the trial (Cohen *d*>1.9). For both groups, medium effect sizes were observed from baseline to after the trial (Cohen *d*=0.2-0.6). Changes over time are shown in Figure 2. Generalized estimating equations analyses revealed statistically significant interaction effects on the primary measures: TYDQ-15 (Wald *χ*²_8_)=152.2; *P*<.001), PHQ-9 (Wald *χ*²_8_=84.2; *P*<.001), and GAD-7 (Wald *χ²*_8_=156.7; *P*<.001). Between-group differences were observed at weeks 4 and 5 on all primary measures (*P*<.001).

Within-group pairwise comparisons showed that TYDQ-15 scores were significantly lower for the IG in weeks 4 and 5 than in all other weeks (*P*s<.001). Within-group analyses also revealed significant differences for the CG, with TYDQ-15 scores at weeks 7 and 8 and after the trial significantly higher than at week 1 or week 2 of phase A (*P*s<.01). On both the PHQ-9 and GAD-7, scores were significantly higher for the IG at weeks 4 and 5 than at all other weeks (*P*s <.001). For the CG, there was a significant decrease in PHQ-9 scores at weeks 7 and 8 (but not after the trial) compared to weeks 1 or 2 (*P*s<.05). No within-group differences were found for the CG on the GAD-7. These results were replicated using the short-form versions of these measures (PHQ-2 and GAD-2; Multimedia Appendix 6).

### Reliable Change on the TYDQ-15

Reliable changes following restriction and recovery are shown in Table 2. From week 1 to week 5, 96.9% (31/32) of IG participants showed reliable deterioration on the TYDQ-15, compared to 1 (2.9%, 1/35) CG participant. From week 5 to week 9, 96.9% (31/32) of IG participants showed reliable improvement on the TYDQ-15. Overall improvement on the TYDQ-15 from week 1 to week 9 was similar for both groups: 22.9% (8/35) for the CG and 28.1% (9/32) for the IG.

**Table 2:** Reliable changes in continuous variables following restriction and subsequent resumption.

Measures and study groups			Restriction (week 1 to 5), n (%)							Resumption (week 5 to 9), n (%)							Overall change (week 1 to 9), n (%)				
			Reliable deterioration		No change		Reliable improvement		Reliable deterioration			No change		Reliable improvement		Reliable deterioration			No change		Reliable improvement
**Things You Do Questionnaire–15**																					
	CG^a^	1 (2.9)		30 (85.7)		4 (11.4)		2 (5.7)			30 (85.7)		3 (8.6)		1 (2.9)			26 (74.3)		8 (22.9)	
	IG^b^	31 (96.9)		1 (3.1)		0 (0)		0 (0)			1 (3.1)		31 (96.9)		1 (3.1)			22 (68.8)		9 (28.1)	
**Patient Health Questionnaire–9**																					
	CG	0 (0)		30 (85.7)		5 (14.3)		1 (2.9)			32 (91.4)		2 (5.7)		0 (0)			29 (82.9)		5 (14.3)	
	IG	21 (65.6)		11 (34.4)		0 (0)		0 (0)			8 (25)		24 (75)		0 (0)			32 (100)		0 (0)	
**Generalized Anxiety Disorder–7**																					
	CG	0 (0)		34 (97.1)		1 (2.9)		0 (0)			34 (97.1)		1 (2.9)		1 (2.9)			32 (91.4)		2 (5.7)	
	IG	20 (62.5)		12 (37.5)		0 (0)		0 (0)			11 (34.4)		21 (65.6)		0 (0)			32 (100)		0 (0)	
**Satisfaction With Life Scale**																					
	CG	1 (2.9)		31 (88.6)		3 (8.6)		0 (0)			35 (100)		0 (0)		1 (2.9)			30 (85.7)		4 (11.4)	
	IG	13 (40.6)		18 (56.3)		1 (3.1)		0 (0)			15 (46.9)		17 (53.1)		1 (3.1)			26 (81.3)		5 (15.6)	

^a^CG: control group.

^b^IG: intervention group.

### Changes in Symptom Severity Categories

Changes in symptom categories on the PHQ-9 and GAD-7 are shown in Table 3. On the PHQ-9, most participants were in the “healthy or mild” range during phases A and C. However, at the end of phase B, 94.3% (33/35) of CG participants remained in the “healthy or minimal” range on the PHQ-9, compared with 28.1% (9/32) of IG participants; 53.1% (17/32) of IG participants were in the “mild” range, and 18.8% (6/32) were in the “moderate” range. These differences were significantly different (*χ*²_1_=26.4; *P*<.001). On the GAD-7, most IG and CG participants were in the “healthy or minimal” range during phase A and at the end of phase C Table 3).

At the end of phase B, 97.1% (34/35) of CG participants were still in the “healthy or mild” range on the GAD-7, compared with 40.6% (13/32) of IG participants; 43.8% (14/32) of IG participants were in the “mild” range, and 15.6% (5/32) were in the “moderate” range. These differences were significantly different (*χ*²_1_=30.8; *P*<.001).

**Table 3:** Changes in symptom severity categories on the Patient Health Questionnaire–9 (PHQ-9) and Generalized Anxiety Disorder–7 (GAD-7).

Measure and severity category				Phase A (baseline), n (%)				Phase B (restriction), n (%)				Phase C (resumption), n (%)									After the trial, n (%)
				Week 1		Week 2		Week 3		Week 4		Week 5		Week 6		Week 7		Week 8		Week 9	
**PHQ-9**																					
	**Control group^a^**																				
		Healthy	31 (88.6)		30 (85.7)		32 (91.4)		33 (94.3)		33 (94.3)		34 (97.1)		34 (97.1)		34 (97.1)		34 (97.1)		
		Mild	3 (8.6)		3 (8.6)		2 (5.7)		1 (2.9)		2 (5.7)		0 (0)		1 (2.9)		1 (2.9)		1 (2.9)		
		Moderate	1 (2.9)		2 (5.7)		1 (2.9)		1 (2.9)		0 (0)		1 (2.9)		0 (0)		0 (0)		0 (0)		
		Moderate-severe	0 (0)		0 (0)		0 (0)		0 (0)		0 (0)		0 (0)		0 (0)		0 (0)		0 (0)		
		Severe	0 (0)		0 (0)		0 (0)		0 (0)		0 (0)		0 (0)		0 (0)		0 (0)		0 (0)		
	**Intervention group^b^**																				
		Healthy	32 (97)		30 (90.9)		30 (93.8)		12 (37.5)		10 (31.3)		29 (90.6)		31 (96.9)		30 (93.8)		31 (96.9)		
		Mild	1 (3)		3 (9.1)		1 (3.1)		14 (43.8)		16 (50)		3 (9.4)		1 (3.1)		2 (6.3)		1 (3.1)		
		Moderate	0 (0)		0 (0)		0 (0)		3 (9.4)		6 (18.8)		0 (0)		0 (0)		0 (0)		0 (0)		
		Moderate-severe	0 (0)		0 (0)		1 (3.1)		2 (6.3)		0 (0)		0 (0)		0 (0)		0 (0)		0 (0)		
		Severe	0 (0)		0 (0)		0 (0)		1 (3.1)		0 (0)		0 (0)		0 (0)		0 (0)		0 (0)		
**GAD-7**																					
	**Control group**																				
		Healthy	32 (94.3)		33 (94.3)		34 (97.1)		33 (94.3)		34 (97.1)		34 (97.1)		33 (94.3)		32 (91.4)		34 (97.1)		
		Mild	2 (5.7)		2 (5.7)		0 (0)		2 (5.7)		1 (2.9)		1 (2.9)		2 (5.7)		3 (8.6)		1 (2.9)		
		Moderate	1 (2.9)		0 (0)		1 (2.9)		0 (0)		0 (0)		0 (0)		0 (0)		0 (0)		0 (0)		
		Severe	0 (0)		0 (0)		0 (0)		0 (0)		0 (0)		0 (0)		0 (0)		0 (0)		0 (0)		
	**Intervention group**																				
		Healthy	31 (93.9)		30 (90.9)		29 (90.6)		14 (43.8)		13 (40.6)		26 (81.3)		29 (90.6)		30 (93.8)		31 (96.9)		
		Mild	2 (6.1)		3 (9.1)		2 (6.3)		14 (43.8)		14 (43.8)		6 (18.8)		3 (9.4)		2 (6.3)		1 (3.1)		
		Moderate	0 (0)		0 (0)		1 (3.1)		1 (3.1)		5 (15.6)		0 (0)		0 (0)		0 (0)		0 (0)		
		Severe	0 (0)		0 (0)		0 (0)		3 (9.4)		0 (0)		0 (0)		0 (0)		0 (0)		0 (0)		

^a^Control group: n=35 at all time points.

^b^Intervention group: n=33 at weeks 1 and 2, and n=32 from week 3 onward.

### Secondary Measures

The IG showed a large within-group effect size on the SWLS from baseline to the end of restriction (Cohen *d*=−0.82) and from restriction to the posttrial phase (Cohen *d*=1.08). Both groups showed a small overall effect size from baseline to the posttrial phase (Cohen *d*=0.1-0.3). Means, SDs, and effect sizes are shown in Table 1. There was a significant time-by-group effect for the SWLS (Wald *χ*²_3_=30.5; *P*<.001). For the CG, there were no statistically significant within-group changes over time. IG scores were significantly lower at week 5 than at week 1 and significantly higher at week 9 than at week 5.

On the first question of the RQ, there were no group differences in how participants rated their mental health (*χ*²_4_=2.9; *P*=.58) at week 1. By week 5, there was a significant difference between groups (*χ*²_4_=52.8; *P*<.001), with 8.6% (3/35) of the CG reporting slight deterioration, compared to 96.9% (31/32) of the IG reporting that their mental health had deteriorated, either slightly (19/32, 59.4%), moderately (10/32, 31.3%), or greatly (2/32, 6.3%). By week 9, there were no longer any group differences (*χ*²_4_=7.1; *P*=.13), and few participants reported deterioration (4/35, 11.4% in the CG and 1/32, 3.1% in the IG reported slight deterioration).

## Discussion

### Principal Findings

The primary aim of this study was to explore whether systematically restricting and then resuming the TYD actions (“restriction-resumption protocol”) led to an increase and then a decrease in symptoms of depression and anxiety among initially asymptomatic adults. The results supported our hypothesis; that is, during the restriction phase, symptom levels rose rapidly, with only 31% (10/32) and 41% (13/32) of IG participants remaining within the clinically healthy range by the end of the phase, for depression and anxiety respectively, compared to 94% (33/35) and 97% (37/35) of controls. Self-reported life satisfaction and perceived mental health followed similar trajectories, suggesting that the observed effects extended to domains of subjective well-being. These findings demonstrate that routine daily actions can exert a significant influence on mental health outcomes.

The secondary aim of this study was to explore participants’ perceptions of changes in their behavior and mental health. A longitudinal qualitative study describing those results found that IG participants identified that the restriction of TYD actions precipitated their declines in mood, energy, and stability [23]. Conversely, participants reported that resuming these actions facilitated recovery, which coincided with increased personal insights into the importance of their coping strategies for mental health.

### Comparisons With Existing Literature

Overall, these results support and extend the findings from our pilot trial [17]. These results are also consistent with previous research indicating that certain behavioral and cognitive actions affect mental health [9,11], as well as with studies showing that the frequency of performing actions linked to good mental health can be increased by sending people daily prompts [25,26].

We note that there was good adherence to the restriction-resumption protocol, suggesting that this novel methodology is highly acceptable and could be used to further explore mechanisms involved in the development of and recovery from common mental disorders. We also note the emergence of other new and promising methods for experimentally manipulating actions to examine how symptoms develop and how they might be prevented or treated [27], but we stress that all new techniques require replication to confirm their validity and minimize demand characteristics or nocebo effects [28].

### Clinical Implications

Our results indicate that, at least for some people, symptoms of depression and anxiety can emerge from understimulation or restriction of healthy actions and habits. They also indicate that, in such cases, symptoms may resolve once those actions and habits are resumed [14,16]. A clinical implication is that, in addition to assessing a person’s presenting difficulties, symptoms, and possible triggers, clinicians should also assess daily habits and actions and provide behavioral prescriptions [29] to promote activity and support recovery from symptoms of mood and anxiety disorders. We have developed a simple TYD checklist for this purpose, which has already been adopted by health services in Australia and elsewhere (Multimedia Appendix 7). We note that this shares important similarities with behavioral activation models of treatment [30], although the range of actions identified by the TYD is broader than the range typically addressed in behavioral activation models, and the TYD model emphasizes the importance of the frequency of performing these actions.

These results provide further support for a threshold of cognitive and behavioral stimulation necessary for maintaining psychological well-being [8], below which psychological dysfunction emerges, but we acknowledge that this requires systematic empirical investigation. A critical next step in understanding the etiology of psychopathological symptoms is to investigate whether restricting specific actions—or categories of actions—has differential effects.

### Ethical Implications

As in our pilot study [17], we applied strict safeguards to reduce risks associated with the restriction phase of our protocol. Nonetheless, planning and conducting this study was a disquieting experience. Although the trial was conducted without incident, the team was relieved to see the recovery to baseline among IG participants. We strongly recommend that any planned replication of this design adopt the risk management strategies outlined in the study by Titov et al [17], as extending the restriction phase beyond 2 weeks may induce clinically significant anxiety or depression requiring treatment in some participants.

### Strengths and Limitations

Key strengths of this study include the use of an RCT design with a sample size adequate to test the hypotheses in healthy volunteers, as well as low withdrawal and high overall questionnaire completion rates. A key limitation is not knowing whether the deterioration was due to participants restricting the TYD actions or due to other factors. We saw no indication that participants restricted other actions or that helpful actions were replaced with actions that caused the increase in symptoms. Not all IG participants experienced increases in symptoms, possibly due to individual differences. These unanswered questions should be explored in larger and more diverse samples in future dismantling and related studies. An important limitation of the study design noted previously is the possible effect of demand characteristics or nocebo effects [28], which should, if possible, be controlled in future trials. Finally, as with all studies concerned with behavior, these results need to be replicated in other less motivated populations.

### Conclusions

This study further demonstrates that restricting the performance of actions associated with mental well-being triggers symptoms of depression and anxiety and that resuming those actions results in symptoms returning to baseline levels in a nonclinical community-based sample. Results confirm the importance of measuring activities as well as symptoms during mental health assessments and highlight the importance of behavioral prescriptions alongside other interventions. They also highlight the potential of the restriction-resumption method as a procedure for better understanding not only the etiology and mechanisms of some forms of depression and anxiety but also how some individuals may recover. The integration of these findings into established public health literature and practice-based mental well-being initiatives warrants consideration for large-scale implementation. Such actions may help reduce the prevalence of some forms of mental disorder or distress while providing consumers and health professionals with additional treatment options.

## Data Availability

The data used in this study will be made available upon reasonable request.

## Appendix

Multimedia Appendix 1 The Big 5 Checklist (Things You Do Questionnaire-15).

Multimedia Appendix 2 Instructions for restriction phase.

Multimedia Appendix 3 Instructions for resumption phase.

Multimedia Appendix 4 SMS text message content.

Multimedia Appendix 5 Participant demographic characteristics and symptom scores at assessment.

Multimedia Appendix 6 Patient Health Questionnaire-2 and Generalized Anxiety Disorder-2 replication.

Multimedia Appendix 7 Checklist for "Things You Do.".

Multimedia Appendix 8 CONSORT checklist.

## Abbreviations

CG: control group
DSM-IV: Diagnostic and Statistical Manual of Mental Disorders, fourth edition
GAD-7: Generalized Anxiety Disorder–7
IG: intervention group
PHQ-9: Patient Health Questionnaire–9
RCT: randomized controlled trial
RQ: Reflections Questionnaire
SWLS: Satisfaction With Life Scale
TYD: Things You Do
TYDQ-15: Things You Do Questionnaire–15
