# Supplementary material for: Impact of Restriction-Resumption Protocols on Mood and Anxiety in Healthy Adults: Randomized Controlled Trial
Source: JMIR Form Res. 2026 May 20;10:e90532. doi: 10.2196/90532 (PMC13234537; doi:10.2196/90532)
Supplement: Multimedia Appendix 1 [file formative_v10i1e90532_app1.docx]

| **The Big 5 Checklist (TYDQ-15)** | | | | | | | **Name:**  **Date:** |
| --- | --- | --- | --- | --- | --- | --- | --- |
| Think about the past 7 days. For each action below, circle how often you did it. | | | | | | |  |
| **Big 5** | **Action** | **Every day  *(4)*** | **5-6 days *(3)*** | **3-4 days  *(2)*** | **1-2 days *(1)*** | **Never *(0)*** | ***Suggestions*** |
| **1. Meaningful Activities** | I did something enjoyable | 4 | 3 | 2 | 1 | 0 | - Take 10 mins to enjoy a song, a show or time outside. Read. Move. Engage in your hobbies. Play. - *Your ideas:* |
|  | I had something to look forward to | 4 | 3 | 2 | 1 | 0 |  |
|  | I did something that was very satisfying to me | 4 | 3 | 2 | 1 | 0 |  |
| **2. Healthy Thinking** | I kept a realistic perspective on things | 4 | 3 | 2 | 1 | 0 | - Accept that making mistakes is human. Keep perspective. Are you treating yourself with respect? - *Your ideas:* |
|  | I dealt with feelings of frustration or impatience in a healthy way | 4 | 3 | 2 | 1 | 0 |  |
|  | I treated myself with respect | 4 | 3 | 2 | 1 | 0 |  |
| **3. Goals and Plans** | I did something to help me achieve my goals | 4 | 3 | 2 | 1 | 0 | - Commit to simple goals each day. Visualise your ‘ideal’ life and plan simple steps towards it. - *Your ideas:* |
|  | I did something to improve or maintain the quality of my life | 4 | 3 | 2 | 1 | 0 |  |
|  | I did something to help me live my "ideal" life | 4 | 3 | 2 | 1 | 0 |  |
| **4. Healthy Routines** | I went to bed and woke up at a regular time | 4 | 3 | 2 | 1 | 0 | - Set-up a relaxing bedtime routine and get up at a regular time. Stretch regularly. Plan a healthy meal. - *Your ideas:* |
|  | I kept a healthy daily routine | 4 | 3 | 2 | 1 | 0 |  |
|  | I prepared and ate a healthy meal | 4 | 3 | 2 | 1 | 0 |  |
| **5. Social Connections** | I socialised with positive people | 4 | 3 | 2 | 1 | 0 | - Send a quick message to someone you care about. Arrange a walk and talk, coffee or phone catch-up. - *Your ideas:* |
|  | I had a meaningful conversation with someone | 4 | 3 | 2 | 1 | 0 |  |
|  | I talked about my day with a friend or family member | 4 | 3 | 2 | 1 | 0 |  |
|  | **Column Totals** | **= ____** | **= ____** | **= ____** | **= ____** | **= ____** |  |
|  | **My Total Score** | **= (Range: 0-60)** | | | | |  |

|  | | | | |
| --- | --- | --- | --- | --- |
| **What Does Your Score Mean?** | | | | |
| **Colours** | **Score Range** | **Range** | **How Often Are You Doing the Big 5/Week?** | **Comments and Suggestions** |
| 🟢🟢 | **53-60** | **Very Healthy** | **Every day or most days** | You're doing great. Keep up your healthy habits — they're clearly working for you. |
| 🟢🟡 | **38-52** | **Healthy** | **5-6 days** | You're in good shape. See if you can pick up one or two more Big 5 actions each week — you might notice you feel even better. |
| 🟡🟡 | **23-37** | **OK** | **3-4 days** | Treat yourself to more enjoyable things each week, even for a few minutes — and better still, with a friend. Remember, you deserve it. |
| 🟡🔴 | **8-12** | **Challenge** | **1-2 days** | This looks like a difficult time for you. Target the areas in the red zone (see Suggestions column) and do one more a day and then slowly increase. If you can manage this over the next two weeks, you're likely to start feeling better. |
| 🔴 🔴 | **0-7** | **Risk** | **Rarely or never** | We can find ourselves in the Risk range for lots of reasons, including due to changes in our lives — illness, loss, or a major life event. Remember your strengths and try to build on them (see Suggestions column). Reach out to someone you trust, and if you feel stuck, please talk to a health professional. |

**Key References
Bisby, M. A., Jones, M. P., Staples, L., Dear, B., & Titov, N. (2024). Measurement of Daily Actions Associated With Mental Health Using the Things You Do Questionnaire-15-Item: Questionnaire Development and Validation Study. JMIR Formative Research, 8, e57804-e57804.
Titov, N., Dear, B. F., Bisby, M. A., Nielssen, O., Staples, L. G., Kayrouz, R., ... & Karin, E. (2022). Measures of daily activities associated with mental health (Things You Do Questionnaire): development of a preliminary psychometric study and replication study. JMIR Formative Research, 6(7), e38837.**
