# Supplementary material for: Impact of Restriction-Resumption Protocols on Mood and Anxiety in Healthy Adults: Randomized Controlled Trial
Source: JMIR Form Res. 2026 May 20;10:e90532. doi: 10.2196/90532 (PMC13234537; doi:10.2196/90532)
Supplement: Multimedia Appendix 2 [file formative_v10i1e90532_app2.pdf]

THE

Things You Do.

PHASE 2: Restriction

You are welcome to contact us at any time during the study:

Professor Nick Titov
Email: contact@ecentreclinic.org
Phone: 0488 991 122

Thank you for participating in this clinical trial of **The Things You Do** model of mental health.

In Phase 2, the Restriction Phase, we want you to restrict how often you do the following actions. This will help us understand the impact of doing these actions on your psychological health.

Please read the descriptions of each of these, below, and then complete the Self-Assessment Sheet over the page.

In Phase 2, we want you to to restrict how often you do these activities each week:

1 Meaningful Activities.

Actions which give us a sense of joy, accomplishment and satisfaction. They can be small things, like listening to a favourite song or watching a good show, and they are often fun to do.

2 Healthy Thinking.

Having realistic thoughts about ourselves, the world and the future. This means keeping perspective and treating ourselves with respect and kindness, particularly when things are difficult.

3 Goals and Plans.

These energise and motivate us. Planning gives us something to look forward to and stops us from dwelling on past problems.

4 Healthy Routines.

These are the things we do automatically, like going to sleep and waking up at the same time, which set us up for the day. Other important routines include those linked to our roles and relationships.

5 Social Connections.

Staying bonded to our family, friends or tribe. Regular contact with people we love and value helps us feel validated/ part of a community.

THE
Things You Do.

PHASE 2:
Restriction

Self-Assessment Sheet.

- This Self-Assessment Sheet contains five types of activities. We have listed three examples for each of these areas.
- Tick the column which best matches how often you did this type of activity in the past week.
- If any of your answers are in the green or yellow columns, please try to **REDUCE** how often you do those activities this week so that your answers are in the red column next week (review the 'Suggestions' column to get some ideas about how you might **REDUCE** them).
- Remember, Phase 2 only lasts for two weeks. Please contact us at any time if you would like to move to Phase 3 (Recovery Phase).

	ACTIVITIES	EXAMPLES	HOW OFTEN DID YOU DO THESE LAST WEEK?					SUGGESTIONS
			Every day	5–6 times per week	3–4 times per week	1–2 times per week	Never	
1	Meaningful Activities.	I did something enjoyable						Spend less time doing the things that you usually enjoy doing, such as listening to music, watching a show, etc.
		I had something to look forward to						Spend less time doing the hobbies and activities that you usually enjoy.
		I did something that was very satisfying to me						Do less of the activities that give you joy and satisfaction.
2	Healthy Thinking.	I kept a realistic perspective on things						Be less flexible and less grounded.
		I dealt with feelings of frustration or impatience in a healthy way						Allow yourself to feel frustrated and annoyed by trivial things.
		I treated myself with respect						Be less self-compassionate or respectful.
3	Goals and Plans.	I did something to help me live my "ideal" life						Make fewer goals or plans this week.
		I did something to help me achieve my goals						Use your online calendar or planner less often this week.
		I did something to improve or maintain the quality of my life						Focus on the past and on the here and now, rather than on your future.
4	Healthy Routines.	I went to bed and woke up at a regular time						Go to bed and get up at a regular time less often than you usually would.
		I kept a healthy daily routine						Do less of your healthy daily routines.
		I prepared and ate a healthy meal						Eat fewer healthy meals.
5	Social Connections.	I socialised with positive people						Spend less time with the people you care about.
		I had a meaningful conversation with someone						Talk to the people in whom you usually confide in less than you usually would.
		I talked about my day with a friend or family member						Talk to loved ones about your day or week less than usual.
