# Supplementary material for: Impact of Restriction-Resumption Protocols on Mood and Anxiety in Healthy Adults: Randomized Controlled Trial
Source: JMIR Form Res. 2026 May 20;10:e90532. doi: 10.2196/90532 (PMC13234537; doi:10.2196/90532)
Supplement: Multimedia Appendix 3 [file formative_v10i1e90532_app3.pdf]

THE

Things You Do.

PHASE 3: Recovery

You are welcome to contact us at any time during the study:

Professor Nick Titov
Email: contact@ecentreclinic.org
Phone: 0488 991 122

Research has shown there are five types of actions that are strongly linked to good mental health. We call these '**The Things You Do**' and we know that doing them regularly can help us thrive and bounce back from challenges.

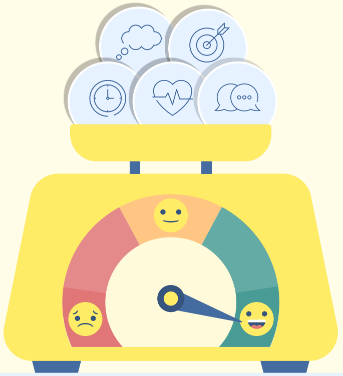

THE MORE OFTEN YOU DO **THE THINGS YOU DO**, THE BETTER YOUR MENTAL HEALTH

Welcome to Phase 3, the Recovery Phase. Please increase how often you do these five types of activities, listed below:

1 Meaningful Activities.

Actions which give us a sense of joy, accomplishment and satisfaction. They can be small things, like listening to a favourite song or watching a good show, and they are often fun to do.

2 Healthy Thinking.

Having realistic thoughts about ourselves, the world and the future. This means keeping perspective and treating ourselves with respect and kindness, particularly when things are difficult.

3 Goals and Plans.

These energise and motivate us. Planning gives us something to look forward to and stops us from dwelling on past problems.

4 Healthy Routines.

These are the things we do automatically, like going to sleep and waking up at the same time, which set us up for the day. Other important routines include those linked to our roles and relationships.

5 Social Connections.

Staying bonded to our family, friends or tribe. Regular contact with people we love and value helps us feel validated/ part of a community.

THE

Things You Do.

PHASE 3: Recovery

Self-Assessment Sheet.

- This Self-Assessment Sheet contains five types of activities, which are strongly linked to mental health.
- We have listed three examples for each of these areas.
- Tick the column which best matches how often you did this type of activity in the past week.
- If any of your answers are in the yellow or red column, please try to **INCREASE** how often you do those activities this week so that your answers are in the green column next week, or at least back to your usual levels (review the 'Suggestions' column to get some ideas about how you might do them **MORE often**).

	ACTIVITIES	EXAMPLES	HOW OFTEN DID YOU DO THESE LAST WEEK?					SUGGESTIONS
			Every day	5–6 times per week	3–4 times per week	1–2 times per week	Never	
1	Meaningful Activities.	I did something enjoyable						Take at least 10 minutes each day to enjoy a favourite piece of music, a TV show, nature or a book.
		I had something to look forward to						Make a list of the simple things you used to enjoy doing and start to re-engage with that hobby or activity.
		I did something that was very satisfying to me						Find activities that align with your values, including doing things for the community, friends, family, pets, or environment.
2	Healthy Thinking.	I kept a realistic perspective on things						Practice accepting that making mistakes is normal and not a sign of weakness.
		I dealt with feelings of frustration or impatience in a healthy way						Treat your frustration as a signal to solve or address the problem that is triggering the situation.
		I treated myself with respect						Check, are you treating yourself in the same way that you would treat others?
3	Goals and Plans.	I did something to help me live my “ideal” life						Visualise your “ideal” life and then plan to do something simple that will help you to start to achieve your vision.
		I did something to help me achieve my goals						Use an online calendar or notebook to help remind you of your goals and plans.
		I did something to improve or maintain the quality of my life						Make a commitment each day to do something simple that will help improve or maintain your quality of life.
4	Healthy Routines.	I went to bed and woke up at a regular time						Create a relaxing bedtime routine to help you settle at night and get up at a regular time each morning.
		I kept a healthy daily routine						Start to take a short (or longer) walk each day.
		I prepared and ate a healthy meal						Add a bit more fruit and veggies to your daily diet.
5	Social Connections.	I socialised with positive people						Make a list of the people you care about, then select three you will talk to each week.
		I had a meaningful conversation with someone						Think about what you want to talk about and to whom.
		I talked about my day with a friend or family member						Make a regular time each week to talk to someone you care about.
