# Supplementary material for: Impact of Restriction-Resumption Protocols on Mood and Anxiety in Healthy Adults: Randomized Controlled Trial
Source: JMIR Form Res. 2026 May 20;10:e90532. doi: 10.2196/90532 (PMC13234537; doi:10.2196/90532)
Supplement: Multimedia Appendix 4 [file formative_v10i1e90532_app4.pdf]

SMS Templates

Day 1

What brings you joy? Do at least one thing today that makes you feel good! Your daily actions matter.

Day 2

Make time for yourself this week. Plan something simple and enjoyable that you can look forward to. We all need goals.

Day 3

Healthy habits start with a good routine. Plan out your day so you can get things done, but also have time for what makes you feel good. Routines matter.

Day 4

Everyone gets busy. Take a few minutes today to text or call that person you have been missing. Connections matter.

Day 5

Instead of being self-critical, congratulate yourself on your efforts - no matter how small. Keep your thoughts healthy.

Day 8

Is there something you want to get done this week? Pop a time in the diary and make it happen! We all need goals.

Day 9

Let yourself laugh every day. Find a joke or a funny meme and have a giggle. Your daily actions matter.

Day 10

It’s time to go easy on yourself. No one is perfect – so don’t expect yourself to be. Keep your thoughts healthy.

Day 11

Sleep helps keep us healthy – physically and mentally. Try and go to bed early at least twice this week. Routines matter.

Day 12

Unwind and de-stress today by calling a friend or relative to chat about how your day went. Connections matter.

Day 15

What activity do you find satisfying and meaningful? Even it’s as simple as a chore, make time for it today. Your daily actions matter.

Day 16

Be your own number one supporter. Nurture and encourage yourself, always. Keep your thoughts balanced.

Day 17

Reach out to a friend today and ask them about their day. Be a listening ear and support them. Caring and sharing makes you stronger. Connections matter.

Day 18

We are what we eat. So, add an extra piece of fruit and veg to your meals today and tomorrow, and feel your health improve! Routines matter.

Day 19

What is something you want to achieve in the next 12 months? Break it down into simple steps and add them to your diary. We all need goals.

Day 22

Let's get active a couple of times this week! Get up and move around for at least 10 minutes – even if it's just a walk around the block. Routines matter.

Day 23

Do you have a favourite hobby? Or want to try out a new one? Let's do it today! Your daily actions matter.

Day 24

Is there someone you've been missing? Call or text that person today and reconnect with them. Connections matter.

Day 25

When you find yourself dwelling on the past, focus on your future. Replace self-defeating thinking with self-encouragement. Keep your thoughts balanced.

Day 26

Planning our weekly schedules can help reduce daily overwhelm. Map out your week so you can get things done and have time to relax. We all need goals.
