# Supplementary material for: Impact of Restriction-Resumption Protocols on Mood and Anxiety in Healthy Adults: Randomized Controlled Trial
Source: JMIR Form Res. 2026 May 20;10:e90532. doi: 10.2196/90532 (PMC13234537; doi:10.2196/90532)
Supplement: Multimedia Appendix 5 [file formative_v10i1e90532_app5.docx]

Table S1. Participant demographic characteristics and symptom scores at assessment

|  | **Control Group**  **(N = 35)** | | **Intervention Group**  **(N = 33)** | | **Significance** |
| --- | --- | --- | --- | --- | --- |
|  | n | % | n | % |  |
| Gender |  |  |  |  |  |
| Male  Female | 11  24 | 31.4%  68.6% | 7  26 | 21.2%  78.8% | *χ²* = .911, *p* = .340 |
| Relationship status |  |  |  |  |  |
| Single  In a relationship  Married/de facto  Divorced/Separated | 13  6  14  2 | 37.1%  17.1%  40.0%  5.7% | 11  5  15  2 | 33.3%  15.2%  45.5%  6.1% | *χ²* = .233, *p* = .972 |
| Highest education qualification |  |  |  |  |  |
| High school  Trade certificate or apprenticeship  University degree or diploma | 11  3  21 | 31.4%  8.6%  60.0% | 1  1  31 | 3.0%  3.0%  93.9% | *χ²* = 11.207,  *p* < .01** |
| Place of residence |  |  |  |  |  |
| Capital city or surrounding suburbs  Other urban location  Rural or remote region | 31  3  1 | 88.6%  8.6%  2.9% | 27  3  3 | 81.8%  9.1%  9.1% | *χ²* = 1.218, *p* = .544 |
| Employment status |  |  |  |  |  |
| Employed full-time  Employed part-time  Not seeking work  Unemployed, seeking work | 12  8  14  1 | 34.3%  22.9%  40.0%  2.9% | 19  8  6  0 | 57.6%  24.2%  18.2%  0.0% | *χ²* = 5.727, *p* = .126 |
| Current difficulties with anxiety |  |  |  |  |  |
| No  Very mild difficulties  Mild difficulties | 29  5  1 | 82.9%  14.3%  2.9% | 25  6  2 | 75.8%  18.2%  6.1% | *χ²* = .662, *p* = .718 |
| Current difficulties with depression |  |  |  |  |  |
| No | 35 | 100.0% | 33 | 100.0% | - |
| Psychotropic medication |  |  |  |  |  |
| No | 35 | 100.0% | 33 | 100.0% | - |
| Currently receiving mental health treatment? |  |  |  |  |  |
| No | 35 | 100.0% | 33 | 100.0% | - |
| Ever consulted a mental health professional? |  |  |  |  |  |
| No  Yes | 30  5 | 85.7%  14.3% | 29  4 | 87.9%  12.1% | *χ²* = .792, *p* = .539 |
|  | M | SD | M | SD |  |
| Age | 34.7 | 13.2 | 37.4 | 9.4 | *F* = .897, *p* = .347 |
| TYD-15 at application | 48.3 | (9.6) | 48.6 | (7.5) | *F* = .028, *p* = .868 |
| PHQ-9 at application | 1.0 | (1.6) | 1.1 | (1.5) | *F* = .157, *p* = .693 |
| GAD-7 at application | 0.6 | (1.2) | 0.5 | (1.1) | *F* = .235, *p* = .629 |
| SWLS at application | 27.5 | (3.6) | 27.3 | (5.5) | *F* = .058, *p* = .811 |

** Significant at p<.01. Data shown as mean and SD for continuous variables and N and % for categorical variables. TYD-15: Things You Do Questionnaire – 15 Item; PHQ-9: Patient Health Questionnaire – 9 Item; GAD-7: Generalized Anxiety Disorder – 7 Item; SWLS: Satisfaction with Life Scale.
