# Supplementary material for: Impact of Restriction-Resumption Protocols on Mood and Anxiety in Healthy Adults: Randomized Controlled Trial
Source: JMIR Form Res. 2026 May 20;10:e90532. doi: 10.2196/90532 (PMC13234537; doi:10.2196/90532)
Supplement: Multimedia Appendix 6 [file formative_v10i1e90532_app6.docx]

**Supplement E**

Replication of results using PHQ-2 and GAD-2.

| Means (SDs) | | | | | |  |  |  |  | Cohen’s *d* within-group effect sizes [95% CIs] | | |
| --- | --- | --- | --- | --- | --- | --- | --- | --- | --- | --- | --- | --- |
|  | Week 1 | Week 2 | Week 3 | Week 4 | Week 5 | Week 6 | Week 7 | Week 8 | Week 9 | Week 1 to Week 5 | Week 5 to Week 9 | Week 1 to Week 9 |
| PHQ-2 |  |  |  |  |  |  |  |  |  |  |  |  |
| CG | 0.4  (0.8) | 0.3  (0.7) | 0.4  (0.8) | 0.3  (0.8) | 0.3  (0.6) | 0.2  (0.7) | 0.2  (0.5) | 0.3  (0.6) | 0.2  (0.5) | -0.14  [-0.63, 0.35] | -0.18  [-0.67, 0.31] | -0.30  [-0.79, -0.20] |
| IG | 0.3  (0.6) | 0.2  (0.5) | 0.4  (0.9) | 1.9  (1.3) | 1.9  (1.1) | 0.6  (0.8) | 0.3  (0.6) | 0.3  (0.6) | 0.2  (0.5) | 1.81  [1.20, 2.36] | -1.99  [-2.56, -1.37] | -0.18  [-0.67, 0.31] |
| GAD-2 |  |  |  |  |  |  |  |  |  |  |  |  |
| CG | 0.2  (0.5) | 0.3  (0.6) | 0.4  (0.8) | 0.3  (0.6) | 0.3  (0.6) | 0.3  (0.7) | 0.2  (0.5) | 0.3  (0.7) | 0.2  (0.5) | 0.18  [-0.31, 0.67] | -0.18  [-0.67, 0.31] | -0.00  [-0.49, 0.49] |
| IG | 0.2  (0.5) | 0.4  (0.7) | 0.4  (0.7) | 1.6  (1.5) | 1.6  (1.1) | 0.8  (0.8) | 0.3  (0.6) | 0.4  (0.7) | 0.3  (0.6) | 1.64  [1.05, 2.18] | -1.47  [-2.00, -0.90] | 0.18  [-0.31, 0.67] |

PHQ-2: Patient Health Questionnaire – 2 Item; GAD-2: Generalized Anxiety Disorder – 2 Item; Control Group (n = 35); IG: Intervention Group (n = 33). One participant withdrew from the Intervention Group at week 2 due to illness. Means substitution was used to replace their missing scores from week 3 onwards. Within group effect sizes are shown as Cohen’s d and 95% conﬁdence intervals.

- - PHQ-2 (Wald’s χ² = 111.6, p < .001)
  - GAD-2 (Wald’s χ²= 19.8, p < .05)
