# Supplementary material for: Impact of Restriction-Resumption Protocols on Mood and Anxiety in Healthy Adults: Randomized Controlled Trial
Source: JMIR Form Res. 2026 May 20;10:e90532. doi: 10.2196/90532 (PMC13234537; doi:10.2196/90532)
Supplement: Multimedia Appendix 7 [file formative_v10i1e90532_app7.pdf]

Supplement A.

Things You Do (Big 5) – 15 (TYD-15)

Please find below a list of activities that are associated with emotional health and wellbeing. Please indicate how often you did each of the following in the last week.

The rating scale is as follows:

0 = Not at all   1 = 1-2 days/week   2 = 3-4 days/week   3 = 5-6 times/week   4 = Every day

1	I kept a healthy daily routine	0	1	2	3	4
2	I went to bed and woke up at a regular time	0	1	2	3	4
3	I treated myself with respect	0	1	2	3	4
4	I dealt with feelings of frustration or impatience in a health way	0	1	2	3	4
5	I socialised with positive people	0	1	2	3	4
6	I talked about my day with a friend or family member	0	1	2	3	4
7	I had a meaningful conversation with someone	0	1	2	3	4
8	I had something to look forward to	0	1	2	3	4
9	I prepared and ate a healthy meal	0	1	2	3	4
10	I did something that was very satisfying to me	0	1	2	3	4
11	I kept a realistic perspective on things	0	1	2	3	4
12	I did something to help me achieve my goals	0	1	2	3	4
13	I did something to improve or maintain the quality of my life	0	1	2	3	4
14	I did something to help me live my "ideal" life	0	1	2	3	4
15	I did something enjoyable	0	1	2	3	4

Reference: Bisby, M. A., Jones, M. P., Staples, L., Dear, B., & Titov, N. (2024). Measurement of Daily Actions Associated With Mental Health Using the Things You Do Questionnaire–15-Item: Questionnaire Development and Validation Study. *JMIR Formative Research*, *8*, e57804.

Supplement B.

THE
Things You Do.

PHASE 2:
Restriction

You are welcome to contact us at any time during the study:

Professor Nick Titov
Email: contact@ecentreclinic.org
Phone: 0488 991 122

Thank you for participating in this clinical trial of **The Things You Do** model of mental health.

In Phase 2, the Restriction Phase, we want you to restrict how often you do the following actions. This will help us understand the impact of doing these actions on your psychological health.

Please read the descriptions of each of these, below, and then complete the Self-Assessment Sheet over the page.

In Phase 2, we want you to to restrict how often you do these activities each week:

1 Meaningful Activities.

Actions which give us a sense of joy, accomplishment and satisfaction. They can be small things, like listening to a favourite song or watching a good show, and they are often fun to do.

2 Healthy Thinking.

Having realistic thoughts about ourselves, the world and the future. This means keeping perspective and treating ourselves with respect and kindness, particularly when things are difficult.

3 Goals and Plans.

These energise and motivate us. Planning gives us something to look forward to and stops us from dwelling on past problems.

4 Healthy Routines.

These are the things we do automatically, like going to sleep and waking up at the same time, which set us up for the day. Other important routines include those linked to our roles and relationships.

5 Social Connections.

Staying bonded to our family, friends or tribe. Regular contact with people we love and value helps us feel validated/ part of a community.

THE
Things You Do.

PHASE 2:
Restriction

Self-Assessment Sheet.

- This Self-Assessment Sheet contains five types of activities. We have listed three examples for each of these areas.
- Tick the column which best matches how often you did this type of activity in the past week.
- If any of your answers are in the green or yellow columns, please try to **REDUCE** how often you do those activities this week so that your answers are in the red column next week (review the 'Suggestions' column to get some ideas about how you might **REDUCE** them).
- Remember, Phase 2 only lasts for two weeks. Please contact us at any time if you would like to move to Phase 3 (Recovery Phase).

ACTIVITIES	EXAMPLES	HOW OFTEN DID YOU DO THESE LAST WEEK?					SUGGESTIONS
		Every day	5–6 times per week	3–4 times per week	1–2 times per week	Never	
1 Meaningful Activities.	I did something enjoyable						Spend less time doing the things that you usually enjoy doing, such as listening to music, watching a show, etc.
	I had something to look forward to						Spend less time doing the hobbies and activities that you usually enjoy.
	I did something that was very satisfying to me						Do less of the activities that give you joy and satisfaction.
2 Healthy Thinking.	I kept a realistic perspective on things						Be less flexible and less grounded.
	I dealt with feelings of frustration or impatience in a healthy way						Allow yourself to feel frustrated and annoyed by trivial things.
	I treated myself with respect						Be less self-compassionate or respectful.
3 Goals and Plans.	I did something to help me live my "ideal" life						Make fewer goals or plans this week.
	I did something to help me achieve my goals						Use your online calendar or planner less often this week.
	I did something to improve or maintain the quality of my life						Focus on the past and on the here and now, rather than on your future.
4 Healthy Routines.	I went to bed and woke up at a regular time						Go to bed and get up at a regular time less often than you usually would.
	I kept a healthy daily routine						Do less of your healthy daily routines.
	I prepared and ate a healthy meal						Eat fewer healthy meals.
5 Social Connections.	I socialised with positive people						Spend less time with the people you care about.
	I had a meaningful conversation with someone						Talk to the people in whom you usually confide in less than you usually would.
	I talked about my day with a friend or family member						Talk to loved ones about your day or week less than usual.

Supplement C.

THE Things You Do.

PHASE 3: Recovery

You are welcome to contact us at any time during the study:

Professor Nick Titov
Email: contact@ecentreclinic.org
Phone: 0488 991 122

Research has shown there are five types of actions that are strongly linked to good mental health. We call these '**The Things You Do**' and we know that doing them regularly can help us thrive and bounce back from challenges.

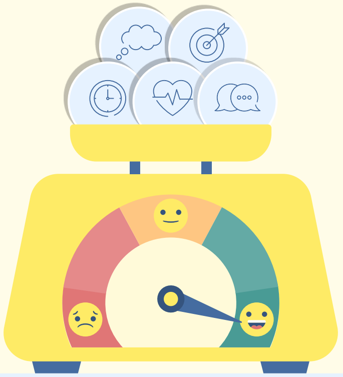

THE MORE OFTEN YOU DO **THE THINGS YOU DO**, THE BETTER YOUR MENTAL HEALTH

Welcome to Phase 3, the Recovery Phase. Please increase how often you do these five types of activities, listed below:

1 Meaningful Activities.

Actions which give us a sense of joy, accomplishment and satisfaction. They can be small things, like listening to a favourite song or watching a good show, and they are often fun to do.

2 Healthy Thinking.

Having realistic thoughts about ourselves, the world and the future. This means keeping perspective and treating ourselves with respect and kindness, particularly when things are difficult.

3 Goals and Plans.

These energise and motivate us. Planning gives us something to look forward to and stops us from dwelling on past problems.

4 Healthy Routines.

These are the things we do automatically, like going to sleep and waking up at the same time, which set us up for the day. Other important routines include those linked to our roles and relationships.

5 Social Connections.

Staying bonded to our family, friends or tribe. Regular contact with people we love and value helps us feel validated/ part of a community.

THE
Things You Do.

PHASE 3: Recovery

Self-Assessment Sheet.

- This Self-Assessment Sheet contains five types of activities, which are strongly linked to mental health.
- We have listed three examples for each of these areas.
- Tick the column which best matches how often you did this type of activity in the past week.
- If any of your answers are in the yellow or red column, please try to **INCREASE** how often you do those activities this week so that your answers are in the green column next week, or at least back to your usual levels (review the 'Suggestions' column to get some ideas about how you might do them **MORE often**).

	ACTIVITIES	EXAMPLES	HOW OFTEN DID YOU DO THESE LAST WEEK?					SUGGESTIONS
			Every day	5–6 times per week	3–4 times per week	1–2 times per week	Never	
1	Meaningful Activities.	I did something enjoyable						Take at least 10 minutes each day to enjoy a favourite piece of music, a TV show, nature or a book.
		I had something to look forward to						Make a list of the simple things you used to enjoy doing and start to re-engage with that hobby or activity.
		I did something that was very satisfying to me						Find activities that align with your values, including doing things for the community, friends, family, pets, or environment.
2	Healthy Thinking.	I kept a realistic perspective on things						Practice accepting that making mistakes is normal and not a sign of weakness.
		I dealt with feelings of frustration or impatience in a healthy way						Treat your frustration as a signal to solve or address the problem that is triggering the situation.
		I treated myself with respect						Check, are you treating yourself in the same way that you would treat others?
3	Goals and Plans.	I did something to help me live my "ideal" life						Visualise your "ideal" life and then plan to do something simple that will help you to start to achieve your vision.
		I did something to help me achieve my goals						Use an online calendar or notebook to help remind you of your goals and plans.
		I did something to improve or maintain the quality of my life						Make a commitment each day to do something simple that will help improve or maintain your quality of life.
4	Healthy Routines.	I went to bed and woke up at a regular time						Create a relaxing bedtime routine to help you settle at night and get up at a regular time each morning.
		I kept a healthy daily routine						Start to take a short (or longer) walk each day.
		I prepared and ate a healthy meal						Add a bit more fruit and veggies to your daily diet.
5	Social Connections.	I socialised with positive people						Make a list of the people you care about, then select three you will talk to each week.
		I had a meaningful conversation with someone						Think about what you want to talk about and to whom.
		I talked about my day with a friend or family member						Make a regular time each week to talk to someone you care about.

Supplement D.

SMS Templates

Day 1

What brings you joy? Do at least one thing today that makes you feel good! Your daily actions matter.

Day 2

Make time for yourself this week. Plan something simple and enjoyable that you can look forward to. We all need goals.

Day 3

Healthy habits start with a good routine. Plan out your day so you can get things done, but also have time for what makes you feel good. Routines matter.

Day 4

Everyone gets busy. Take a few minutes today to text or call that person you have been missing. Connections matter.

Day 5

Instead of being self-critical, congratulate yourself on your efforts - no matter how small. Keep your thoughts healthy.

Day 8

Is there something you want to get done this week? Pop a time in the diary and make it happen! We all need goals.

Day 9

Let yourself laugh every day. Find a joke or a funny meme and have a giggle. Your daily actions matter.

Day 10

It’s time to go easy on yourself. No one is perfect – so don’t expect yourself to be. Keep your thoughts healthy.

Day 11

Sleep helps keep us healthy – physically and mentally. Try and go to bed early at least twice this week. Routines matter.

Day 12

Unwind and de-stress today by calling a friend or relative to chat about how your day went. Connections matter.

Day 15

What activity do you find satisfying and meaningful? Even it’s as simple as a chore, make time for it today. Your daily actions matter.

Day 16

Be your own number one supporter. Nurture and encourage yourself, always. Keep your thoughts balanced.

Day 17

Reach out to a friend today and ask them about their day. Be a listening ear and support them. Caring and sharing makes you stronger. Connections matter.

Day 18

We are what we eat. So, add an extra piece of fruit and veg to your meals today and tomorrow, and feel your health improve! Routines matter.

Day 19

What is something you want to achieve in the next 12 months? Break it down into simple steps and add them to your diary. We all need goals.

Day 22

Let's get active a couple of times this week! Get up and move around for at least 10 minutes – even if it's just a walk around the block. Routines matter.

Day 23

Do you have a favourite hobby? Or want to try out a new one? Let's do it today! Your daily actions matter.

Day 24

Is there someone you've been missing? Call or text that person today and reconnect with them. Connections matter.

Day 25

When you find yourself dwelling on the past, focus on your future. Replace self-defeating thinking with self-encouragement. Keep your thoughts balanced.

Day 26

Planning our weekly schedules can help reduce daily overwhelm. Map out your week so you can get things done and have time to relax. We all need goals.

Supplement E.

Replication of results using PHQ-2 and GAD-2.

	Means (SDs)									Cohen's *d* within-group effect sizes [95% CIs]		
	Week 1	Week 2	Week 3	Week 4	Week 5	Week 6	Week 7	Week 8	Week 9	Week 1 to Week 5	Week 5 to Week 9	Week 1 to Week 9
**PHQ-2**												
CG	0.4 (0.8)	0.3 (0.7)	0.4 (0.8)	0.3 (0.8)	0.3 (0.6)	0.2 (0.7)	0.2 (0.5)	0.3 (0.6)	0.2 (0.5)	-0.14 [-0.63, 0.35]	-0.18 [-0.67, 0.31]	-0.30 [-0.79, -0.20]
IG	0.3 (0.6)	0.2 (0.5)	0.4 (0.9)	1.9 (1.3)	1.9 (1.1)	0.6 (0.8)	0.3 (0.6)	0.3 (0.6)	0.2 (0.5)	1.81 [1.20, 2.36]	-1.99 [-2.56, -1.37]	-0.18 [-0.67, 0.31]
**GAD-2**												
CG	0.2 (0.5)	0.3 (0.6)	0.4 (0.8)	0.3 (0.6)	0.3 (0.6)	0.3 (0.7)	0.2 (0.5)	0.3 (0.7)	0.2 (0.5)	0.18 [-0.31, 0.67]	-0.18 [-0.67, 0.31]	-0.00 [-0.49, 0.49]
IG	0.2 (0.5)	0.4 (0.7)	0.4 (0.7)	1.6 (1.5)	1.6 (1.1)	0.8 (0.8)	0.3 (0.6)	0.4 (0.7)	0.3 (0.6)	1.64 [1.05, 2.18]	-1.47 [-2.00, -0.90]	0.18 [-0.31, 0.67]

PHQ-2: Patient Health Questionnaire – 2 Item; GAD-2: Generalized Anxiety Disorder – 2 Item; Control Group (n = 35); IG: Intervention Group (n = 33). One participant withdrew from the Intervention Group at week 2 due to illness. Means substitution was used to replace their missing scores from week 3 onwards. Within group effect sizes are shown as Cohen's d and 95% confidence intervals.

- PHQ-2 (Wald's χ^2 = 111.6, p < .001)
- GAD-2 (Wald's χ^2= 19.8, p < .05)

Supplement F.

The Big 5

THINGS YOU CAN DO FOR
BETTER MENTAL HEALTH

Our research with over 20,000 people has shown that five types of actions are strongly linked to good mental health.

We call these 'The Big 5' and we know that people who do them regularly each week are likely to have good mental health.

Key Points:

- Doing The Big 5 regularly is the foundation for good mental health.
- Most people stop doing The Big 5 when they feel unwell, stressed, or experience a change in their lives.
- Use The Big 5 Checklist on the next page to check your Big 5 activity and to get ideas about how to do them more often.
- If after two weeks you aren't improving, please visit your GP or MindSpot for professional support.

The Big 5 are things we can all do. They are the building blocks for good mental health and include:

1 **Meaningful Activities.** These give us a sense of accomplishment, satisfaction or joy. They can be as simple as listening to a favourite song or watching a good show, and they are often fun and engaging.

2 **Healthy Thinking.** This means having realistic thoughts about ourselves, the world, and the future. This also means keeping perspective and treating ourselves with respect, particularly when things are difficult.

3 **Goals and Plans.** Having a goal or a plan helps us stay motivated and energised. Goals give us something to look forward to and stop us dwelling on past problems.

4 **Healthy Routines.** Routines we do automatically like our sleep routine, eating well or being active, are critical for good mental health. These actions set us up for the day.

5 **Social Connections.** Regular contact with people we love and respect, which may include our family, friends or tribe, help us to feel validated and give us a sense of belonging.

REMEMBER THIS SIMPLE BIG 5 RULE:

'DO MORE AND FEEL BETTER, DO LESS AND FEEL WORSE'

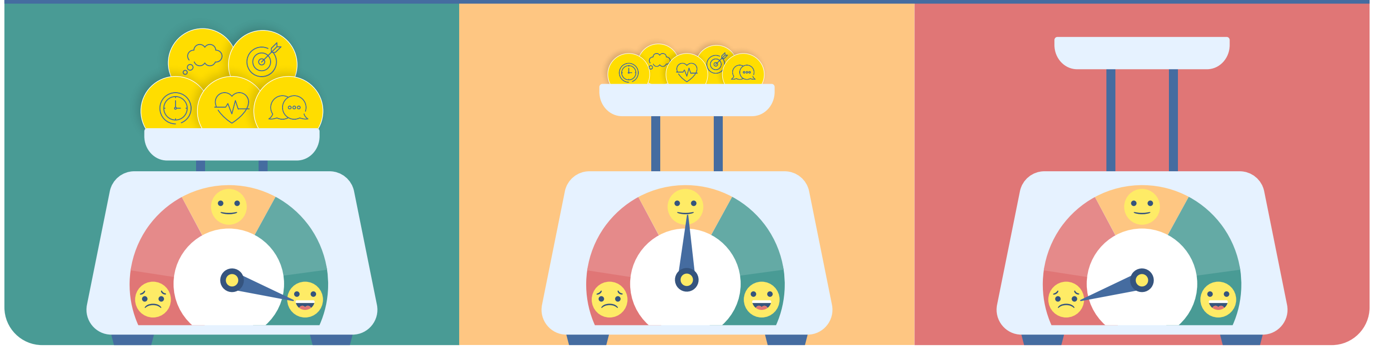

© MindSpot 2024

The Big 5 Self-Assessment Checklist

MindSpot

To learn more about MindSpot, please contact us:
contact@mindspot.org.au
1800 61 44 34
mindspot.org.au

- This Self-Assessment Checklist contains five types of activities, which are strongly linked to mental health
- Tick the column which best matches how often you did this type of activity in the past week
- We have listed three examples for each of these areas
- If any of your answers are in the yellow or red section, check the next column to get some ideas about how you might do them more often

ACTIVITIES	EXAMPLES	HOW OFTEN DID YOU DO THESE EACH WEEK?					SUGGESTIONS
		Every day	5–6 times per week	3–4 times per week	1–2 times per week	Never	
1 Meaningful Activities.	I did something enjoyable	◯	◯	◯	◯	◯	Take at least 10 minutes each day to enjoy a favourite piece of music, a show, time outside or a book
	I had something to look forward to	◯	◯	◯	◯	◯	Make a list of the simple things you used to enjoy doing and start to re-engage with that hobby or activity
	I did something that was very satisfying to me	◯	◯	◯	◯	◯	Find activities that align with your values, including doing things for the community, friends, family, pets, or environment
2 Healthy Thinking.	I kept a realistic perspective on things	◯	◯	◯	◯	◯	Practice accepting that making mistakes is normal and not a sign of weakness
	I dealt with feelings of frustration or impatience in a healthy way	◯	◯	◯	◯	◯	Treat your frustration as a signal to solve or address the problem that is triggering your reactions
	I treated myself with respect	◯	◯	◯	◯	◯	Check, are you treating yourself in the same way that you would treat others?
3 Goals and Plans.	I did something to help me live my "ideal" life	◯	◯	◯	◯	◯	Visualise your "ideal" life and then plan to do something simple that will help you to start to achieve your vision
	I did something to help me achieve my goals	◯	◯	◯	◯	◯	Use an online calendar or notebook to help remind you of your goals and plans
	I did something to improve or maintain the quality of my life	◯	◯	◯	◯	◯	Make a commitment each day to do something simple that will help improve or maintain your quality of life
4 Healthy Routines.	I went to bed and woke up at a regular time	◯	◯	◯	◯	◯	Create a relaxing bedtime routine to help you settle at night and get up at a regular time each morning
	I kept a healthy daily routine	◯	◯	◯	◯	◯	Start to take a short (or longer) walk each day
	I prepared and ate a healthy meal	◯	◯	◯	◯	◯	Add a bit more fruit and veggies to your daily diet
5 Social Connections.	I socialised with positive people	◯	◯	◯	◯	◯	Make a list of the people you care about, then select three you will talk to each week
	I had a meaningful conversation with someone	◯	◯	◯	◯	◯	Think about what you want to talk about and to whom
	I talked about my day with a friend or family member	◯	◯	◯	◯	◯	Make a regular time each week to talk to someone you care about

MS_The-Big-5_DIGITAL_V11_241204
